# Next Generation Sequencing for Diagnosis of Leptospirosis Combined With Multiple Organ Failure: A Case Report and Literature Review

**DOI:** 10.3389/fmed.2021.756592

**Published:** 2022-01-25

**Authors:** Juan Lu, Juan Hu, Shanshan Yu, Lanjuan Li

**Affiliations:** ^1^State Key Laboratory for Diagnosis and Treatment of Infectious Diseases, National Clinical Research Center for Infectious Diseases, Collaborative Innovation Center for Diagnosis and Treatment of Infectious Diseases, The First Affiliated Hospital, College of Medicine, Zhejiang University, Hangzhou, China; ^2^State Key Laboratory for Diagnosis and Treatment of Infectious Diseases, Intensive Care Unit, College of Medicine, The First Affiliated Hospital, Zhejiang University, Hangzhou, China

**Keywords:** leptospirosis, next generation sequencing, diagnosis, multiple organ failure, clinical treatment

## Abstract

**Introduction:**

Leptospirosis poses a major threat to human life. The disease spectrum ranges from a nearly undetectable presentation to severe multi-organ dysfunction and death. Leptospirosis is difficult to diagnose by traditional antibody and culture tests. We here present a case of multiple organ failure associated with leptospirosis.

**Material and Methods:**

A 64-year-old woman presented with fatigue and arthralgia, which developed rapidly into multiple organ injuries, and she eventually died of cerebral hemorrhage. Serum antibody test and cultures of blood, sputum, urine, and feces samples were all negative. The patient was diagnosed with leptospirosis by the next-generation sequencing (NGS).

**Conclusion:**

We conclude that leptospirosis is a neglected zoonosis caused by pathogenic *Leptospira* species. New techniques such as NGS are highlighted for early diagnosis. Surveillance for pathogens during diagnosis can provide guidance for clinical treatment and improves prognosis.

## Introduction

Leptospirosis is a widely distributed zoonosis and a major public health problem. Its clinical manifestations are complex and diverse; misdiagnosis is common and the fatality rate is high (1). Humans can be infected via direct or indirect contact with animal urine or excreta, making them unintentional and susceptible hosts. Leptospirosis ranges from a mild and self-limiting illness to severe disease and death (2). Early detection, accurate diagnosis, timely treatment, and close observation are essential to improve cure rate and decrease fatality rate (3). The classical bacterial culture process requires a long time (about 13 weeks). Moreover, the antibody detection has poor specificity and tends to yield false positive results (4, 5). New techniques including nucleic acid molecular diagnostic technologies, such as polymerase chain reaction (PCR) or high-throughput sequencing using next-generation sequencing (NGS) have been developed and applied in the detection of leptospirosis, which can provide valuable guidance for clinical treatment (5–8). Here, we present a case of leptospirosis caused by *Leptospira* interrogans associated with symptomatic multi-organ dysfunction.

## Case Presentation

On October 13, 2020, a 64-year-old woman was referred to our hospital with fatigue, low blood pressure, arthralgia, and myalgia, for 1 week. She had a history of untreated hydronephrosis for 4 years and was positive for hepatitis B surface antigen for several decades. She did farm work in the mountain without personal protection 1 week prior to admission to a local hospital.

Her laboratory findings on admission were as follows: white blood cell (WBC) count 15.3 × 10^9^/L; platelet (PLT) level 10 × 10^9^/L; albumin level 22.7 g/L; alanine transaminase (ALT) level 79 U/L; aspartate aminotransferase (AST) level, 134 U/L; total bilirubin (TBI) level 105.3 μmol/L; direct bilirubin (DBI) level 60.6 μmol/L; creatinine level 87.3 μmol/L; blood urea nitrogen level 16.68 mmol/L; prothrombin time (PT), 18.6 s; activated partial thromboplastin time (APTT) 41.9 s; and D-dimer level 6,320 μg/L. Her blood pressure (120/73 mmHg) and breathing was stable.

Four days later, her blood pressure fell dramatically to 84/58 mmHg, jaundice appeared, and renal function deteriorated despite symptomatic treatment. Subsequently, she was transferred to our hospital and complained of generalized pain without fever and jaundice on the entire body. Physical examination revealed thickened wet rales in both lungs, abdominal distention, and edema in both lower limbs. Neurological examination was negative. Other signs were normal. The Glasgow Coma Scale score was 15/15 and blood pressure was 100/80 mmHg and pulse rate 98 beats/min (on noradrenaline maintenance) in the supine position. Laboratory parameters were markedly elevated: white blood cell count 57.0 x 10^9^/L; total bilirubin 254.5 μmol/L; creatinine 263 μmol/L; procalcitonin 10.73 ng/mL; and C-reactive protein 59.3 mg/L. Chest radiography revealed obvious patchy shadows in both lungs. Supportive care included infusion of thrombocytes and plasma, liver protection, and relief of jaundice by high-flow oxygen therapy (60 L/min). Under an initial diagnosis of pneumonia, meropenem (1.0 g three times per day) was prescribed in combination with continuous renal replacement therapy.

**Figure 1:**
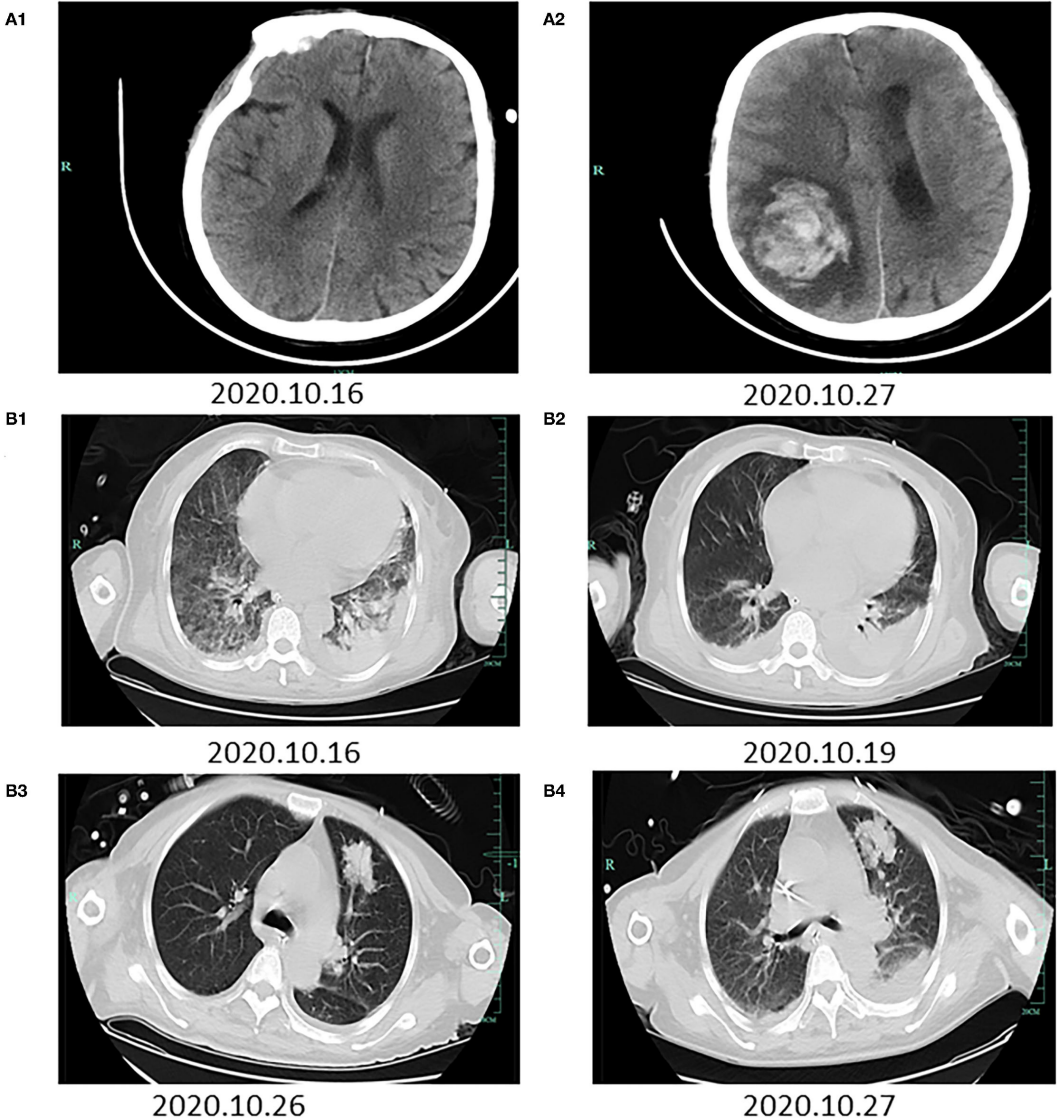
Radiographic images during the hospitalization. The first row of brain CTs **(A1,A2)** was taken on October 16 and 27, 2020, respectively. **(A1)** A normal brain. **(A2)** Hemorrhage in the right paraoccipital lobe that had ruptured the lateral ventricle. The second and third row of chest CTs **(B1–B4)** was taken on October 16, 19, 26, and 27, 2020, respectively. **(B1)** Bilateral patchy shadows and diffuse infiltration and extensive consolidation in the right lung. **(B2)** Hydrothorax and relief of bilateral exudation. **(B3)** New patchy shadows in the right upper lung. **(B4)** Patchy shadows in the right upper lung and bilateral exudation.

On days 2–4 of hospitalization, the patient's condition worsened, and we commenced noninvasive assisted ventilation under high-flow oxygen (60 L/min). The patient's maximum body temperature was 37.2°C. Tracheoscopy revealed extensive hemorrhage in both airways. Brain computed tomography (CT) findings were normal (Figure 1A1). Chest CT revealed bilateral patchy shadows and diffuse infiltration and extensive consolidation in the right lung (Figure 1B1). Etiological cultures of blood, sputum, urine, and feces samples were performed; serum antibodies against dengue, leptospirosis, and epidemic hemorrhagic fever were tested for, considering the patient's history of farm work in the mountains. Bone marrow puncture was performed due to obviously abnormal blood routine examinations. Samples of sputum, urine, and feces were collected to detect *Leptospira* IgM and IgG antibodies, which were both negative. Multidisciplinary diagnosis and treatment were carried out by the Departments of Hematology, Infectious Disease, Pneumology, and Organ Transplantation, as well as the Intensive Care Unit. PCR was conducted using the diagnostic reagent kits based on the guidelines of the Center for Disease Control (CDC) of Zhejiang Province. Next-generation sequencing (NGS) of blood and sputum samples was also conducted. Supportive organ therapy was initiated. The patient developed multiple organ failure, but the cause was unclear. Entecavir (25 mg per day) and ornithine aspartate (40 mg per day) were administered to lower the high blood ammonia level. Her liver cirrhosis with ascites was evident on abdominal CT, possibly due to the patient's long history of hepatitis B surface antigen-positivity.

NGS revealed *Leptospira interrogans* (four different sequences of leptospires DNA, Supplementary Material 1) in blood on day 4 of hospitalization. Two family members who had engaged in farm work also underwent antibody test for leptospirosis, which revealed negative results. Her red blood cell count was decreased, and the free iron level was very high; chronic anemia was apparent. Meropenem was terminated and penicillin was initiated (four injections of 800,000 units per day) combined with dexamethasone (20 mg per day) to prevent the Hector reaction. Repeat chest CT indicated hydrothorax and relief of bilateral exudation (Figure 1B2).

On days 5–13 of hospitalization, The test of bone marrow puncture showed no obvious abnormality. However, the patient's liver function deteriorated; she was sedated under non-invasive assisted ventilation and administered continuous renal replacement therapy. Considering the severe jaundice and inflammatory response caused by the pulmonary infection and activation of hepatitis B virus, as well as liver failure, plasmapheresis was performed to remove inflammatory factors and improve liver function on days 8, 9, and 10. Piperacillin-tazobactam (4.5 g three times per day) was commenced because *Pseudomonas aeruginosa* was detected in sputum on day 10. Further sputum cultures revealed *Aspergillus fumigatus*; chest CT revealed improvement of the infiltrates in both lungs except for new patchy shadows in the right upper lung (Figure 1B3). Voriconazole (200 mg twice a day) was prescribed as antifungal therapy. The priorities at this time were symptomatic treatments including venous nutritional support, adjustment of the acid-base balance and circulatory parameters, prevention of gastrointestinal bleeding, and protection of organ parenchymae. On day 14, the patient suddenly vomited blood (200 mL) and then lost consciousness; her Glasgow Coma Scale score was suddenly decreased to 3/15. Emergency brain CT revealed hematoceles in both lateral ventricles and hemorrhage in the right paraoccipital lobe that had ruptured the lateral ventricle (Figure 1A2). Chest CT was also repeated (Figure 1B4). We communicated with the family members about the patient's condition and explained that no further treatment options were available. The patient died 5 h later.

## Discussion

Leptospirosis is sporadic in the temperate regions around the world. It is an epidemic-prone disease carrying the causative agents in the damaged mucous membranes, and is generally caused by exposure to contaminated water. The clinical manifestations of leptospirosis are complex and easily mimic other life-threatening infections, and diagnosis and differential diagnosis are particularly important. Our patient initially presented with fatigue and arthralgia, followed by rapid multiple organ damage, which rendered early diagnosis difficult. It is crucial to perform early antibody and culture tests. Liver failure was obvious, suggesting the presence of jaundice, coagulation defect, and history of hepatitis B-associated liver cirrhosis. However, other diseases should also be considered because of the abnormal blood tests, poor renal function, and subsequent multiple organ dysfunction. Although leptospirosis antibody results were negative, the presence of pathogens could not be completely ruled out as leptospires are frequently found in low numbers. Several diagnostic tests are available for leptospirosis including detection of the pathogen and antibodies. These tests are particularly useful in the early stage of this disease and before the use of antimicrobial drugs when bacterial numbers are highest in the blood and urine (9). *Leptospira* culture allows detection of leptospires, but it is technically difficult due to the demand of special growth media (10). Because leptospires are fastidious so as to require enrichment media and long incubation time under *in vitro* conditions independent of infection date, culture is less helpful for clinical diagnosis (11). In the traditional antibody tests, MAT is often used (12). However, identification of the infecting serovar is difficult based on the cross-reactive MAT results on account of laboratory variation and differences in host-specific humoral immune responses (13). Molecular diagnostic tools, such as PCR, have become widely available in commercial diagnostic laboratories for the diagnosis of leptospirosis (14). False-negative results can be encountered if there are low bacterial loads as a result of disease phase, immune response, or administration of antimicrobial drugs (15, 16). Several ELISAs for detecting IgM, IgG, or both antibody types have been developed to detect specific antibodies in leptospirosis. However, its overall sensitivity is lower compared with the MAT, which has not been previously recommended (17, 18).

Patients may benefit from next-generation sequencing (NGS), which is a method that allows for high-throughput, massively parallel sequencing of thousands to billions of DNA fragments independently and simultaneously. Over the past five years, NGS testing has been used not only in basic research but also in clinical diagnosis, especially are applied in clinical microbiology examinations (19). The NGS testing for our case showed the evidence of four different sequences of leptospires DNA (Supplementary Material 2). NGS in microbiology examinations can reveal the characteristics of pathogens by morphology and a genomic definition of pathogens (20). If only culture is performed, diagnosis of severe and acute infection may be missed, leading to inadequate or delayed treatment, and increased morbidity and mortality (21). Appropriate antimicrobial treatment in combination with supportive therapy would be beneficial to reducing the mortality from this disease. Due to the difficulty in the cultivation or poor growth rate of some organisms, as well as the use of prophylactic antimicrobial drugs, conventional testing methods used in microbiological diagnosis are limited in pathogen detection, thus compromising the effectivity of antimicrobial drugs (22). From this perspective, NGS is a promising and sensitive method to detect the selected organism types and discover early, new or unexpected organisms (23).

Most deaths from leptospirosis are attributed to renal failure and/or gastrointestinal, pulmonary, or cerebral hemorrhage (24–26) caused by extensive capillary damage including extensive renal necrosis and glomerular atrophy. Some bacterial pathogens produce hemolysins including Sph2, and the expression of Sph2 is highest among epithelial cells (27). *L. interrogans* Sph2 was recently found to trigger inflammatory reaction, cytomembranous injury, and apoptosis by damaging the membranes of blood vessel endothelial cells through increasing intracellular reactive oxygen species (ROS) and decreasing the mitochondrial membrane potential (MMP). Moreover, *Leptospira* increases the permeability and engorgement of blood vascular cells (28–30). In addition, platelet aggregation, a crucial player in the blood coagulation process, is inactivated independent of PI3K/AKT-ERK signaling pathways, thereby causing hemorrhage in tissues (31). Activation of coagulation factor-III extends the coagulation time, and is associated with pulmonary, renal, and cerebral hemorrhage, leading to death due to severe types of leptospirosis (32, 33).

## Conclusion

Leptospirosis is a neglected zoonosis caused by pathogenic *Leptospira*. It ranges from a mild and self-limiting illness to severe disease and death. The correct diagnosis could be established by actively adopting new examination methods such as NGS, which should be performed early to avoid misdiagnosis. The NGS technology represents a useful opportunity and tool for precise diagnostics to identify pathogens in infectious diseases.

## Data Availability Statement

The datasets presented in this study can be found in online repositories. The names of the repository/repositories and accession number(s) can be found in the article/Supplementary Material.

## Ethics Statement

Ethical review and approval was not required for the study on human participants in accordance with the local legislation and institutional requirements. The patients/participants provided their written informed consent to participate in this study. Written informed consent was obtained from the individual(s) for the publication of any potentially identifiable images or data included in this article.

## Author Contributions

JL and LL structured the text and content. JL and JH wrote and edited the manuscript and generated the figures. SY reviewed the literature. All authors contributed intellectually to manuscript content, read, and agreed to the published version of the manuscript.

## Funding

This research was funded by the Independent Project Fund of the State Key Laboratory for Diagnosis and Treatment of Infectious Diseases, the National Key Research and Development Program of China (Grant No. 2016YFC1101404/3), and Zhejiang Basic Public Welfare Research Program of China (Grant No. LQ20H030012).

## Conflict of Interest

The authors declare that the research was conducted in the absence of any commercial or financial relationships that could be construed as a potential conflict of interest.

## Publisher's Note

All claims expressed in this article are solely those of the authors and do not necessarily represent those of their affiliated organizations, or those of the publisher, the editors and the reviewers. Any product that may be evaluated in this article, or claim that may be made by its manufacturer, is not guaranteed or endorsed by the publisher.
