# Supplementary material for: Next Generation Sequencing for Diagnosis of Leptospirosis Combined With Multiple Organ Failure: A Case Report and Literature Review
Source: Front Med (Lausanne). 2022 Jan 25;8:756592. doi: 10.3389/fmed.2021.756592 (PMC8821090; doi:10.3389/fmed.2021.756592)
Supplement: Supplementary file 1 [file Data_Sheet_1.PDF]

IngeniSeq®

病原微生物宏基因组测序报告

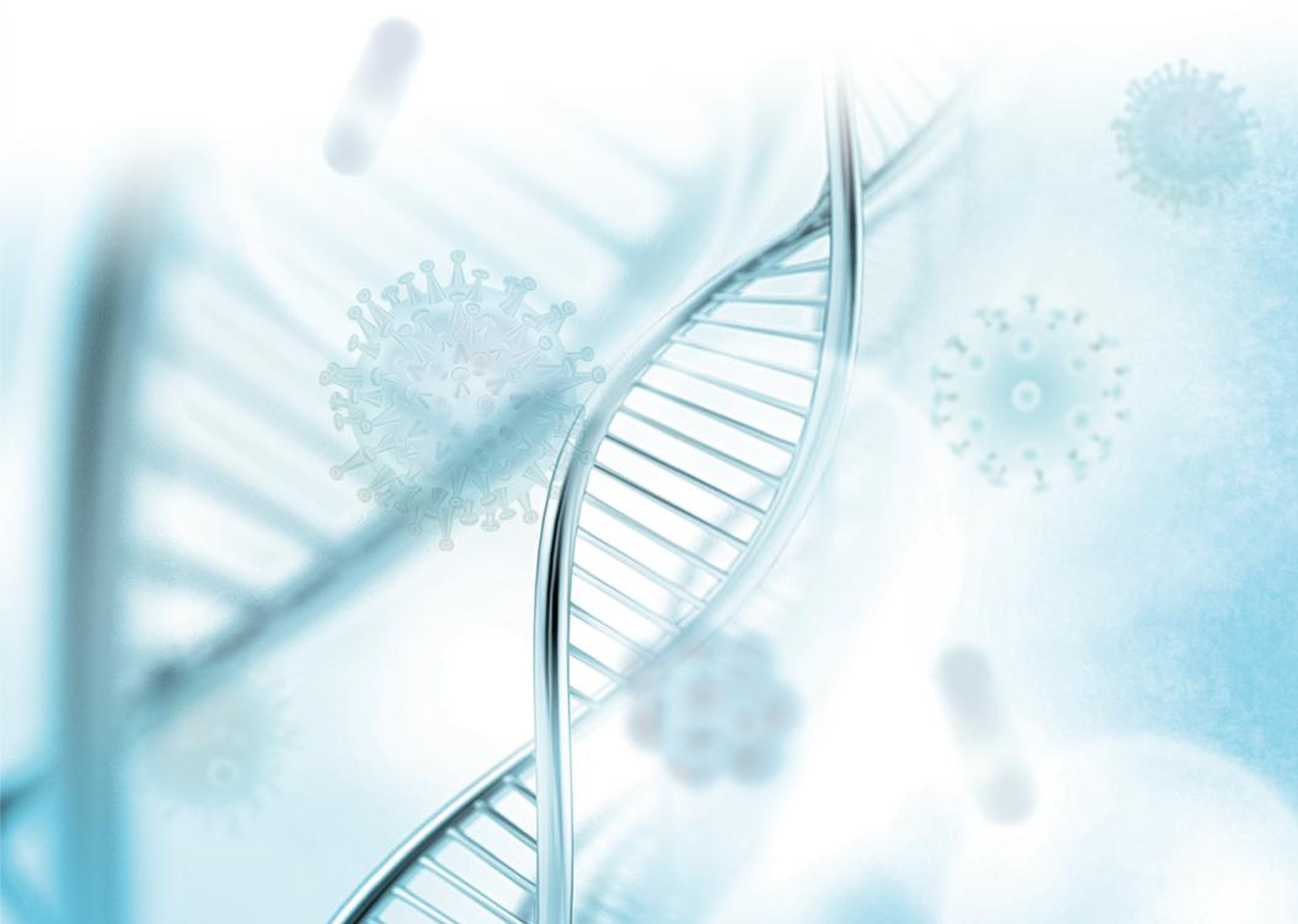

基本信息

**样本编号**	012010150010		**样本类型**	EDTA 抗凝血	
**样本来源**	**医院：**浙江大学医学院附属第一医院（庆春院区） **科室：**ICU **医生：**万婷婷				
**采样时间**	2020.10.14	**收样时间**	2020.10.15	**病案号**	05515626
**受检者姓名**	陈莉英	**性别**	女	**年龄**	64 岁
**临床症状**	乏力 4 天，血压下降 1 天，感染性休克，急性肝功能衰竭，急性呼吸衰竭，肾功能不全，血小板减少，病毒性肝炎				
**检测项目**	病原微生物宏基因组检测-DNA				

**检测结果及临床解读**	**细菌性感染：**问号钩端螺旋体（*Leptospira_interrogans*）

具体检测结果

细菌检测结果		序列总数	备注
问号钩端螺旋体	*Leptospira_interrogans*	4	

真菌检测结果		序列总数	备注
未检出	未检出		

DNA 病毒检测结果		序列总数	备注
未检出	未检出		

RNA 病毒检测结果		序列总数	备注
未检测	未检测		

寄生虫检测结果		序列总数	备注
未检出	未检出		

支原体/衣原体/立克次体检测结果		序列总数	备注
未检出	未检出		

补充报告（仅供参考）

微生物	中文名	序列数	检出类型	备注
Mycolicibacterium_smegmatis	耻垢分枝杆菌	1	细菌	可能环境微生物

注意及声明：

1. 该补充报告显示的结果为已经排除了实验室污染后检测出的所有微生物，其中可能包括：
    1）样本采集过程中或分装过程中受到污染的环境微生物或其核酸；
    2）样本采集容器本身带有的环境微生物或其核酸;
    3）样本采集过程中受到污染的病人身上（皮肤、上呼吸道、口腔、肠道等等）的人体共生微生物；
    4）样本采集过程中收到污染的采集或分装人员身上（皮肤、上呼吸道、口腔、肠道等等）的人体共生微生物；
    5）定植于病人特定身体部位的微生物；
    6）有可能造成真正感染的病原微生物。
2. 该报告无法区别定植微生物与病原微生物。
3. 该报告仅供参考，不能作为临床诊断或用药的唯一依据。

耐药基因

ARO 名字	常见病原体	序列数	覆盖度百分比（碱基数）	药物类型	耐药机制
未检出					

术语解释：

1. ARO 名字：CARD 数据库中的基因名
2. 常见病原体：该基因一般存在的微生物（由于耐药基因转移等，也可能在其它微生物中发现）
3. 序列数：比对上该耐药基因的 reads 数目
4. 覆盖度百分比（碱基数）：该基因被 reads 覆盖的百分比（该基因被 reads 覆盖的碱基个数）
5. 耐药机制：耐药的作用机制

耐药基因检测说明：

1. 本产品通过 reads 比对到耐药基因，通过一定的算法筛选，从而确定样品中是否有这个耐药基因。通过耐药基因功能，从而预测耐药的可能性。耐药基因类型主要包括：外排、修饰、失活、阻遏等。
2. 本产品所使用的耐药数据库为 CARD 数据库（Comprehensive Antibiotic Resistance Database）

耐药检测的局限性：

临床研究表明，耐药基因与实际表型可能不完全一致，报告中的耐药基因检测结果仅供参考。耐药预测局限原因主要由以下几点：

1. 由于病原体宏基因组样品特征，微生物基因组覆盖不全，并不能检测到所有菌的耐药基因，所以很可能会漏检测一些耐药；
2. 由于病原体宏基因组测序深度低，对于一些基因突变所导致的基因失活，不能进行判断，从而导致所检测的耐药有一定的假阳性；

人体菌群微生态

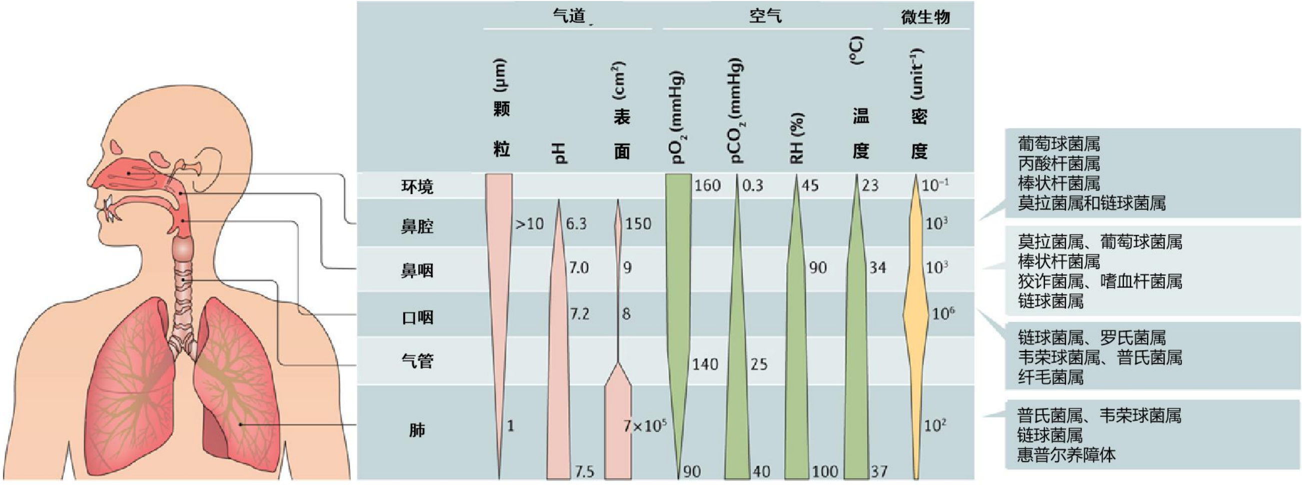

呼吸道微生态是指上下呼吸道中包括细菌、真菌、病毒、支原体、衣原体在内的全部微生物的集合。上呼吸道包括前鼻、鼻腔、鼻窦、鼻咽、口咽和声带上方的喉部。其中前鼻部是最接近外界环境的部位，黏膜下含有大量的浆液和皮脂腺体，容易导致一些亲脂性微生物的定居，如葡萄球菌属、棒状杆菌属、丙酸杆菌属等。此外，前鼻部还含有莫拉氏菌属、狡诈球菌属和链球菌属等呼吸道常见菌属。鼻咽部位于鼻腔深部，比起鼻前庭和鼻腔，鼻咽部具有更加多样化的微生物群落特征。除了含有葡萄球菌属、莫拉氏菌属、棒状杆菌属之外，鼻咽部还含有大量的狡诈球菌属、嗜血杆菌属、链球菌属等。口咽部在呼吸道中微生态多样性、微生物密度最高，含有大量的奈瑟菌属、罗氏菌属和以普氏菌属、韦荣球菌属、纤毛菌属为代表的厌氧菌，链球菌属是口咽部的特征性菌属。下呼吸道则包括声带下方部分喉 部、气管、小支气管、细支气管和肺泡。部分研究认为健康人下呼吸道微生物主要来源于上呼吸道微生物的扩散，因此，下呼吸道存在的菌群大多数能在上呼吸道找到，如莫拉菌属、嗜血杆菌属、葡萄球菌属和链球菌属等。但下呼吸道缺少存在于上呼吸道的棒状杆菌属、狡诈菌属等，这可能与下呼吸道的清除功能有关。研究发现，呼吸道微生物在呼吸道健康中扮演着重要的角色，呼吸道微生态的变化与多种疾病相关，如哮喘、肺囊性纤维化、慢性阻塞性肺疾病等。

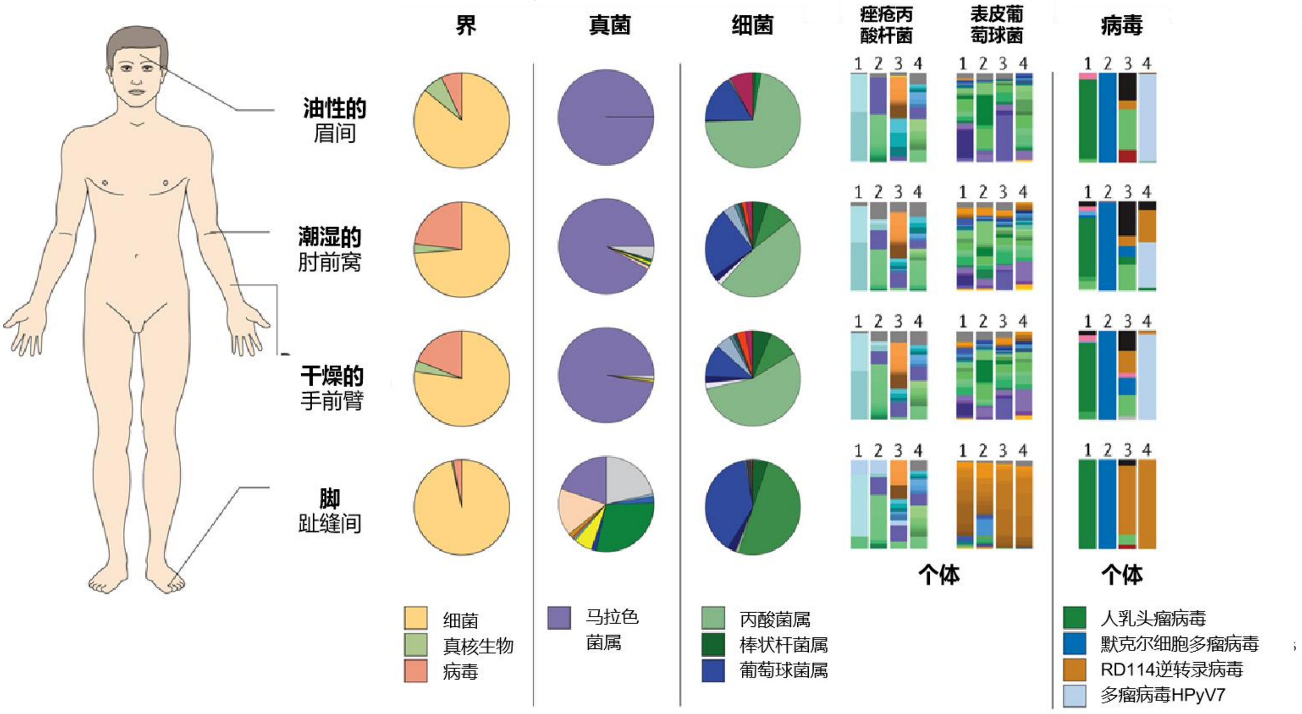

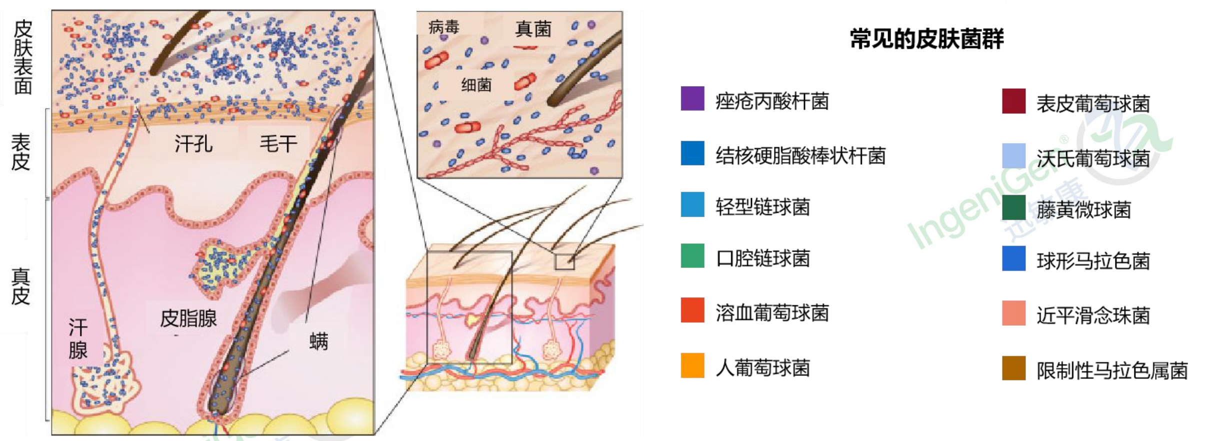

皮肤是机体和环境接触的主要保护屏障，皮肤由两个结构不同的层组成分别是表皮和真皮。在人体皮肤上栖息着数以万计的微生物，其中大多数对人体是无害，甚至是有益的，这些微生物在人体皮肤组成的复杂生态系统被称为皮肤微生态。皮肤微生物群包含有细菌、古生菌、真菌、病毒尤以噬菌体为主等 ，这些生物体皆非孤立存的，是以种群聚集于某个特定栖息部位，称之为生态位。微生物与皮肤的寄居关系，将皮肤微生物群分为常驻微生物和暂驻型微生物常驻微生物群主要存在于皮肤毛囊周围的上表皮，以葡萄球菌、枯草芽孢、微球菌、棒状杆菌属等多见，皮肤微生物群落组成和动态分布与皮肤表面组织细胞之间

存在着整体平衡，这种动态平衡一旦被打破，它们又成为条件致病菌，引起许多感染性疾病，可能会对人体健康产生影响。

人类机体承载着数量庞大的微生物群，各种细菌、病毒、真菌等定居在身体表面，包括消化道、皮肤、口腔、阴道、上呼吸道和肺。这些定植的微生物与机体之间相互作用，构成了人体特定的微生态环境。

检测方法及内容

本检测采用美国illumina公司的高通量测序技术，对样本中微生物核酸序列进行宏基因组分析，与数据库中微生物核酸序列进行比对，结合临床表现，鉴定病原微生物。检测范围包括目前已知的病原体包括细菌库7044 种、DNA 病毒库 9233 种、RNA 病毒库 1309 种、真菌库 2890 种、寄生虫 172 种、支原体 139 种、衣原体 128 种、立克次氏体 102 种和分枝杆菌 635 种。最低检测限为100copies / ml，特异性大于99.6%。

数据处理

以机器学习(machine learning)进行同步式错误探查(error modeling)、背景清除(denoising)与精确序列推理(exact sequence inference)，严格质控系统，自动消除假阳性结果。

临床解析

报告解读

问号钩端螺旋体（*Leptospira_interrogans*）

钩端螺旋体属于螺旋体目螺旋体科钩端螺旋体属,分为两个种:双曲钩端螺旋体(Leptospira biflexa)和问号钩端螺旋体(Leptospira interrogans);前者为腐生性钩端螺旋体,通常对人和动物不致病;后者为寄生性、致病性钩端螺旋体,是引起人和动物钩端螺旋体病的病原体.钩端螺旋体病(Leptospirosis,简称钩体病),是由问号钩端螺旋体引起的一种人兽共患疾病,在世界范围广泛分布,尤其流行于热带及亚热带地区。【1】

参考文献

1.杨会棉 蒋秀高.钩端螺旋体的分子分型方法[J].中国人兽共患病学报.2011, 27(11):1024-1027.

mNGS 方法学参考文献

(1) Chiu, Charles Y., and Steven A. Miller. Clinical metagenomics. *Nat Rev Genet* 20.6 (2019): 341-355.

(2) Gu W, Miller S, Chiu C Y, et al. Clinical Metagenomic Next-Generation Sequencing for Pathogen Detection.[J]. Annual Review of Pathology-mechanisms of Disease, 2019, 14(1): 319-338.

(3) Li H, Gao H, Meng H, et al. Detection of Pulmonary Infectious Pathogens From Lung Biopsy Tissues by Metagenomic Next-Generation Sequencing[J]. Frontiers in Cellular and Infection Microbiology, 2018.

(4) Miller S, Naccache S N, Samayoa E, et al. Laboratory validation of a clinical metagenomic sequencing assay for pathogen detection in cerebrospinal fluid.[J]. Genome Research, 2019, 29(5): 831-842.

(5) Miao Q, Ma Y, Wang Q, et al. Microbiological Diagnostic Performance of Metagenomic Next-generation Sequencing When Applied to Clinical Practice[J]. Clinical Infectious Diseases, 2018.

(6) Vijayvargiya P, Jeraldo P, Thoendel M, et al. Application of metagenomic shotgun sequencing to detect vector-borne pathogens in clinical blood samples[J]. PLOS ONE, 2019, 14(10).

https://card.mcmaster.ca/

http://ccb.jhu.edu/software/kraken/
