# Supplementary material for: Next Generation Sequencing for Diagnosis of Leptospirosis Combined With Multiple Organ Failure: A Case Report and Literature Review
Source: Front Med (Lausanne). 2022 Jan 25;8:756592. doi: 10.3389/fmed.2021.756592 (PMC8821090; doi:10.3389/fmed.2021.756592)
Supplement: Supplementary file 2 [file Data_Sheet_2.PDF]

>@NB551669:519:HFYMNBGXG:1:13310:16324:3664 1:N:0:GGATTAGG
GATTGATACTTTCCGGAAAAAAAACTCCTTCTAGAAATCATCTCGCACAGTCTTACGAACAAGTCCGAGCTTCTAT
>@NB551669:519:HFYMNBGXG:3:22406:20080:13703 1:N:0:GGATTAGG
GTGGAGGATTCTCCTTCTGATTCTTCTAAAATATCGTATCCAGAAGTTCGAATGATCGTAGAAGAATCGATGT
>@NB551669:519:HFYMNBGXG:3:21511:1296:16693 1:N:0:GGATTAGG
CTTTTAATAGAACTGCTCGAAGTGTTTTCTTGAATGTTTGCAAATTGAGATAGTTTTATAAACTCACCCGTTGGAG
>@NB551669:519:HFYMNBGXG:4:23410:25662:20042 1:N:0:GGATTAGG
TCAGAGAGGTAGACGGGGAATTTGATATTTATATCAATGACTAATGGATTCCTAGACTTAAAGTTAATAAAGAATA
